# Knowledge, attitude, and practices on intestinal schistosomiasis among primary schoolchildren in the Lake Victoria basin, Rorya District, north-western Tanzania

**DOI:** 10.1186/s12889-017-4767-9

**Published:** 2017-09-21

**Authors:** David Z. Munisi, Joram Buza, Emmanuel A. Mpolya, Teckla Angelo, Safari M. Kinung’hi

**Affiliations:** 1 Department of Global Health and Bio-Medical Sciences, School of Life Sciences and Bio-Engineering, Nelson Mandela African Institution of Science and Technology, P. O. Box, 447 Arusha, Tanzania; 2 Department of Bio-Medical Sciences, School of Medicine and Dentistry, College of Health Sciences, University of Dodoma, P. O. Box 259, Dodoma, Tanzania; 3 National Institute for Medical Research (NIMR), Mwanza Research Centre, Isamilo Road, P. O. Box 1462, Mwanza, Tanzania

**Keywords:** Schistosomiasis, Knowledge, Attitude, Practices, Schoolchildren, Tanzania

## Abstract

**Background:**

Globally school-age children, adolescents and young adults bear the highest burden of schistosomiasis. When developing a specific intervention to improve community’s knowledge, attitudes, and practices (KAPs), existing KAPs must be taken into account. Therefore, this study was designed to determine schoolchildren’s KAPs on schistosomiasis in the study area.

**Methods:**

A cross-sectional study was conducted in Busanga and Kibuyi villages involving 513 schoolchildren. A pre-tested questionnaire was used to collect socio-demographic data and to assess KAP on schistosomiasis among primary schoolchildren in the study area.

**Results:**

Of the 488 interviewed children, 391 (80.12%) reported to have heard of schistosomiasis, with the majority 289 (73.91%) citing school as the source of this knowledge. Swimming in the lake, worms, witchcraft, and mosquitoes were mentioned to be the cause for intestinal schistosomiasis. Fishing in the lake, drinking unboiled lake water, walking bare footed, and shaking hands were reported to be practices that may lead to contracting schistosomiasis. Only 156 (39.90%) of the study respondents reported to know the signs of intestinal schistosomiasis. Avoiding swimming in the lake, drinking unboiled water and eating unwashed fruits were mentioned as preventive measures. Nearly 85% (412) reported understanding that there was a disease known as schistosomiasis; additionally, 419 (85.86%) considered schistosomiasis as a dangerous disease and 418 (85.66%) believed that schistosomiasis was treatable. Fishermen and schoolchildren were reported to be groups most at risk of schistosomiasis infection. Visiting the lake (for swimming and other gatherings) was a common practice among study participants 471 (96.52%).Nearly 93% (451) of participants mentioned using lake water for domestic chores, and, although 407 (84.61%) reported to own a toilet at home, only 229 (55.31%) reported to always use a toilet for sanitation purposes.

**Conclusion and recommendation:**

Despite a high rate of awareness among schoolchildren regarding schistosomiasis, there was a persistent gap amongst the children regarding the causes, modes of transmission, symptoms, and preventive measures for the disease. Therefore, an appropriate health education intervention is needed in order to inculcate better knowledge, attitudes, and practices amongst schoolchildren regarding its transmission, control, and prevention as part of a successful schistosomiasis campaign.

## Background

Schistosomiasis (also known as bilharzias) is a neglected tropical disease (NTD) of public health importance in many tropical and sub-tropical developing countries [1]. The disease occurs in 74 countries worldwide, with an annual estimate of 207 million people being infected globally and an additional 779 million people at risk of infection. Sub-Saharan Africa (SSA) accounts for more than 90% of the cases [2, 3]. In Tanzania schistosomiasis is highly prevalent and the country ranks second, after Nigeria, in terms of disease burden on the African continent [2, 4, 5]. Intestinal schistosomiasis caused by *Schistosoma mansoni* (*S. mansoni*) is highly endemic in areas surrounding Lake Victoria in Tanzania [4, 6], and is implicated in significant morbidity [4, 7]. School-aged children, adolescents, and young adults are groups that bear the highest burden of disease resulting into significant impairment of their physical, nutritional, and cognitive potentials [3, 4, 8–10].

Three key approaches are currently used to control schistosomiasis, including: improved sanitation, health education, and mass treatment with Praziquantel [11]. However, in many endemic areas including Tanzania, schistosomiasis control has largely relied on periodic mass treatment of school-age children with Praziquantel in accordance with World Health Organization (WHO) recommendations [8, 12]. Lack of awareness about the modes of transmission of parasitic infections increases the risk of infection and therefore re-infection following treatment [13]. Moreover, in high transmission settings, if there has been no change in the sanitary practices and exposure patterns, re-infection tends to occur within one year following treatment, with such trends being higher among young children and adolescents as their adult counterparts demonstrate an acquired partial resistance to re-infections following treatment [14, 15].

When trying to develop specific interventions aiming at improving communities’ KAPs, pre-existing capacities must be taken into account [16]. This baseline KAPs level will inform bridging of identified gaps to enhance successful disease control [11]. Health promotion interventions are likely to fail if they are designed without understanding the typical health behaviours of the target population [16]. Furthermore, for interventions focusing on community awareness and involving low socioeconomic communities, it is recommended to create supportive environment for the success and sustainability of other strategies [17, 18].

Although schistosomiasis is prevalent in areas surrounding the Victoria Lake Basin in Tanzania, information on the KAPs on the disease of the most at-risk groups is scarce in the public domain. Therefore, this study was designed to determine schoolchildren’s KAPs on schistosomiasis in the study area.

## Methods

### Study area

This study was conducted in Rorya District in north-western Tanzania. The district is bordered by Tarime District to the east, Butiama District to the south, Lake Victoria to the west, and the Republic of Kenya to the north [19]. For a more detailed description of the study area please see Munisi et al. [20].

### Study design

This study was a cross-sectional baseline survey that assessed knowledge, attitudes, and practices on schistosomiasis among primary schoolchildren in selected schools in the study area.

### Study population, inclusion and exclusion criteria

The study population was comprised of primary schoolchildren aged 6–16 years attending pre-grade one to grade six in Busanga and Kibuyi primary schools in the two villages of Busanga and Kibuyi, respectively. Busanga primary school had a total of 690 pupils, of whom 337 were boys and 353 girls while Kibuyi primary school had 737 pupils of whom, 366 were boys and 371 girls. Schoolchildren aged between 6 and 16 years, who gave assent to participate in the study and whose parents/guardians provided a written informed consent were eligible for the study. Schoolchildren with a history of being clinically ill and used anti-schistosome drugs within a period of six months before the study, were excluded as described in Munisi et al. [20].

### Sample size determination and sampling procedures

This report reflects on a baseline survey nested within a longitudinal interventional study aimed at comparing cure and eggs reduction rates for two different treatment regimens for intestinal schistosomiasis using Praziquantel. Sample size was calculated using a formula for comparing two rates [21]. In the calculations we used cure rates reported from a study of communities living along the shores of Lake Albert in Uganda, which reported cure rates of 41.9% and 69.1% for single dose and two doses treatment regimen, respectively [22]. The level of significance was set at 95% and power of 90%. Adding 30% annual loss to follow up, a total sample size of 257 per treatment group was required, but we managed to recruit a total of 513 study participants for the entire study.

We conveniently selected two schools within two villages namely Busanga and Kibuyi that lies along the shore of Lake Victoria. We then randomly selected 246 and 267 schoolchildren from Busanga and Kibuyi primary schools, respectively. We sampled children from pre-grade one to grade six. We excluded children in grade seven because they were about to do their final national examinations and they would have been absent during the subsequent follow-up surveys. The number of schoolchildren selected from each class was determined by the probability proportional to the number of children in each class. We attempted to sample equal numbers of boys and girls from each class. Systematic random sampling method was used to obtain study participants for each sex from each class. The schoolchildren in each class were requested to stand in two lines, one for boys and the other one for girls and they were counted. The sampling interval was obtained by dividing the total number of each sex in the class with the number of each sex to be investigated from that class (N/n). After obtaining a starting point from a table of random numbers, children were sampled according to the sampling interval. The same interval was repeated until the required number of children for each sex in each class was obtained as described in our previous publication [20].

### Data collection

#### Assessment of socio-demographic information and risk factors

A pre-tested Swahili translated semi-structured questionnaire was used to gather information on demographic characteristics of the study participants and their KAPs towards *S. mansoni* infection. Variables such as age, sex, socio-economic activities of parents/guardians, sanitary practices, water contact behaviour and history of receiving anti-schistosomal treatment were assessed. Also, the questionnaire involved questions concerning the knowledge about schistosomiasis aetiology, transmission, clinical manifestations, prevention, and control. The questionnaire was initially developed in English, then translated to Swahili, and then back-translated by a different person who was blinded to the original questionnaire.

#### Data analysis

The collected data were entered into a database using EpiData Version 3.1. Data analysis was done using STATA Version 12.1 (Stata Corp, Texas, USA). Descriptive statistics, including percentages and mean values, were used to summarize the data. The chi-square test was used to assess the association between categorical variables. *P*-values less than 0.05 were considered statistically significant.

#### Ethical statement

The Medical Research Coordination Committee (MRCC) of the National Institute for Medical Research (NIMR), Tanzania provided an approval for this study (Reference number NIMR/HQ/R.8a/Vol. IX/1990). The study received further approval from the District Executive Director, District Education Officer, and Medical Officer of Rorya District Council. Prior to the study, the research team conducted meetings with the village executive officers, teachers, and students of selected villages and schools, respectively. During these meetings, the objectives of the study, the study procedures, sampling, study benefits, and potential risks and discomforts were explained. Informed consent for all children who participated in the study was sought from parents or legal guardians through an informed consent form. Assent was sought from children who were also informed of their rights to refuse to participate in the study and to withdraw from the study at any time. At baseline, all children were given a standard dose of praziquantel (40 mg/kg) and albendazole (400 mg) as a single dose after stool sample collection. Treatment with praziquantel was given after a meal which was prepared and offered at school to minimize potential side effects. Treatment was performed under direct observation (DOT) of a qualified nurse.

## Results

### Socio-demographic characteristics of the study participants

A total of 513 schoolchildren from the two primary schools were enrolled in the study. Out of these, 488 (95.13%) completed the interview. Of the interviewed children 238 (48.77%) were from Busanga village and 250 (51.23%) were from Kibuyi village. Among the study participants, 244 (50.00%) were males and the other 244 (50.00%) were females. The numbers of boys and girls in Busanga primary school were 117 (49.16%) and 121 (50.84%), respectively whereas the numbers of boys and girls in Kibuyi primary school were 127 (50.80%) and 123 (49.20%), respectively. The age of participating schoolchildren ranged from 6 to 16 years with a mean age of 10.97 ± 2.36 years. The distribution of children with 6–9 years were 136 (27.87%), 10–12 years were 208 (42.62%) and 13–16 years were 144 (29.51%).

### Respondent’s knowledge on the cause, transmission, symptoms and preventive measures against *Schistosomiasis*

Of the 488 interviewed children, 391 (80.12%) reported hearing of schistosomiasis, with the majority (289, 73.91%) of the children mentioning school as a source of information regarding schistosomiasis. The majority 339, (86.70%) of the children mentioned swimming in the lake to be the cause of schistosomiasis, while only 44 (11.25%) mentioned that worms cause the disease. Witchcraft was mentioned by 15 (3.84%) of those who reported to have heard about the disease, while 13 (3.32%) mentioned mosquito to be responsible for causing intestinal schistosomiasis (Table 1). Respecting contributory activities for schistosomiasis infection, the majority of the respondents (339, 86.7%) mentioned swimming in the lake and fishing (316, 80.86%). Fewer respondents indicated causes such as drinking unboiled water (251, 64.19%); walking barefooted (220;56.27%) and shaking hands(49, 12.53%) respectively. In terms of knowledge of symptoms for intestinal schistosomiasis, only 156 (39.9%) of the study respondents reported knowing the symptoms for intestinal schistosomiasis, of which 136 (87.18%) mentioned stomach ache. The majority of respondents (306, 78.26%) mentioned avoiding swimming in the lake as a preventive measure for intestinal schistosomiasis, Avoiding drinking unboiled water and washing fruits before eating were also mentioned by more than half the respondents [232 (59.34%); 251 (64.19%)], respectively (Table 1).

**Table 1:** Respondents’ knowledge on the cause, transmission, symptoms and preventive measures for *Schistosomiasis*

Variable	Frequency	Percentage
Ever heard of Schistosomiasis (*n* = 488)	391	80.12
Source of information (*n* = 391)		
School	289	73.91
Home	149	38.11
Local dispensary	53	13.55
News media	93	23.79
Causes of Schistosomiasis (n = 391)		
Schistosomiasis is caused by worms	44	11.25
Schistosomiasis is caused by mosquitoes	13	3.32
Schistosomiasis is caused by witchcraft	15	3.84
Schistosomiasis is caused by swimming in ponds	102	26.09
Schistosomiasis is caused by swimming in river	49	12.53
Schistosomiasis is caused by swimming in lake	339	86.70
I don’t know what causes Schistosomiasis	37	9.46
Transmission of intestinal schistosomiasis		
Activities that may lead to getting Intestinal schistosomiasis (*n* = 391)		
Swimming in the lake	339	86.70
Fishing in the lake	316	80.82
Washing clothes in the lake	251	64.19
Washing dishes in the lake	220	56.27
Drinking unboiled water	267	67.77
Walking barefooted	188	48.08
Shaking hands	49	12.53
Signs for intestinal schistosomiasis (*n* = 156)		
Know the signs for intestinal schistosomiasis	156	39.90
Blood in urine (Haematuria)	81	51.92
Painful urination	40	25.64
Stomach ache	136	87.18
Swelling abdomen	61	39.10
Preventive measures for intestinal schistosomiasis (*n* = 391)		
Avoiding swimming in the lake	306	78.26
Wearing gum boots when in contact with lake water	258	65.98
Always using toilets	289	73.91
Avoiding touching the soil	117	29.92
Washing hands	169	43.22
Avoiding drinking unboiled water	232	59.34
Washing fruits before eating	251	64.19

### Attitude, risk perception and practices of the study participants towards schistosomiasis

**Fig. 1:**
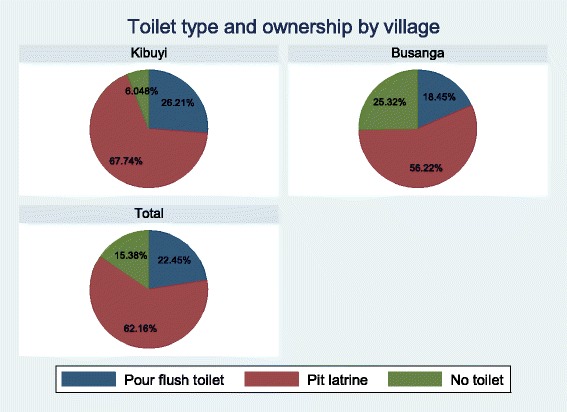
Toilet type and ownership by village of residence

**Fig. 2:**
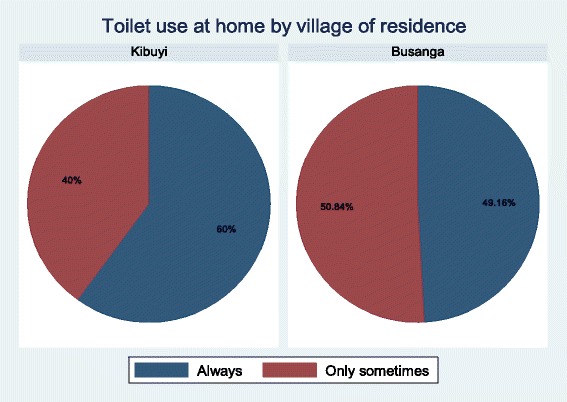
Toilet use at home by village of residence

The majority of the children [412 (84.77%)] understood that there was schistosomiasis in their village of residence. Among the interviewed children, 419 (85.86%) considered schistosomiasis to be a dangerous disease,while 418 (85.66%) understood that the disease can be treated (Table 2). The majority of respondents [354, 81.76%] reported that fishermen were the most at-risk group for intestinal schistosomiasis, schoolchildren were also mentioned to be among the at-risk group by 325 (75.06%) participants (Table 2). The most common source of water used for domestic chores was Lake Victoria water, with 451 (92.42%) of study participants reporting this source (Table 2). Toilet ownership was common with 407 (84.61%) reporting a toilet at home with the main toilet type being a pit latrine [299, 62.16%]. There were significantly more children who reported not to have a toilet at home in Busanga (25.32%) than Kibuyi (6.048%) (Fig. 1) (*p* < 0.001). However, only 229 (55.31%) reported to always use a toilet, while 185 (44.69%) reported to use a toilet only sometimes. Defecating in the bushes was reported by 184 (98.91%) of those who use the toilets only sometimes and 154 (84.15%) reported to also defecate along the lakeshore. Visiting Lake Victoria was a common practice among study participants (471, 96.52%) of which 412 (87.85%) reported daily visits (Table 2). Slightly more than half (50.84%) of the respondents in Busanga village reported using a toilet sometimes, while it was only 40% in Kibuyi village yielding a statistically significant (*p* = 0.028)finding (Fig. 2).

**Table 2:** Attitude, risk perception and practices of the study participants towards schistosomiasis

Variable	Frequency	Percentage (1%)
Source of water used at home (*n* = 488)		
Tap water	39	7.99
Lake water	451	92.42
Bore hole	15	3.07
Open well	51	10.45
River water	16	3.28
Type of toilet at home (*n* = 481)		
Pour flush toilet	108	22.45
Pit latrine	299	62.16
No toilet	74	15.38
Sanitary practices		
Always use toilet (*n* = 414)	229	55.31
Use toilet only sometimes (*n* = 414)	185	44.69
Sometimes defecate along the lake shore (*n* = 183)	154	84.15
Sometimes defecate in the bushes (*n* = 183)	181	98.91
Water contact habits (*n* = 488)		
Visiting the lake	471	96.52
Frequency of visiting the lake (469)		
Once a month	11	2.35
2–3 times a week	46	9.81
Everyday	412	87.85
Risk perception (*n* = 488)		
Schistosomiasis can be treated	418	85.66
There is schistosomiasis where I am living	412	84.77
Schistosomiasis is a dangerous disease	419	85.86
Schistosomiasis is a chronic disease	40	8.20
Schistosomiasis is a shameful disease	4	0.82
Schistosomiasis is not a very dangerous disease	21	4.30
I don’t know	4	0.82
Most at risk groups (*n* = 433)		
School children	325	75.06
Women	58	13.39
Rice farmers	32	7.39
Fishermen	354	81.76

## Discussion

The success of schistosomiasis control interventions in endemic areas can be realized if children, who are the targets of the currently used control interventions, have adequate knowledge, positive attitudes, and correct preventive and control practices. This study aimed at exploring the level of KAPs of schoolchildren on schistosomiasis to inform the development of a targeted control strategy against schistosomiasis.

Our findings showed that majority of the respondents understood that there was schistosomiasis in their village of residence and considered schistosomiasis as a dangerous disease. A similar finding was also reported in other studies elsewhere [11, 13]. The majority also admitted that the disease could be treated, as it was also reported by Mazigo et al. [23].

In this study, it was found that majority of respondents had heard about intestinal schistosomiasis, which is similar to previous study findings [11, 13, 23, 24]. However, just having heard about the disease is insufficient, with a proper understanding of the disease and its causes and mode of transmission required [16]. About three quarters of respondents reported the school to be one of the major sources of information about schistosomiasis, again reflected in previous studies and making schools a potential strategic channel for communicating health information to this most susceptible age group [23, 25, 26]. In contrast, other studies reported the most common source of information about schistosomiasis to be family or neighbours, which may dilute the knowledge leading to various misconceptions [13].

Although the majority of the study participants mentioned swimming in the lake to be one way by which intestinal schistosomiasis could be transmitted, visiting the lake was common in this community. This high rate of visiting the lake was also reported in another study where 84% of the children reported going to the lake [23]. Children also mentioned fishing as an activity through which schistosomiasis might be transmitted. This high level of knowledge on schistosomiasis transmission could be due to the endemic rate in this community. Of greater concern is how this high rate of knowledge has not concomitantly translated into preventive practices [20]. Surprisingly, only a few (11.25%) of the participants knew that the cause of schistosomiasis were worms, which seems to be a common issue reported in other studies [23, 24, 26]. Misconceptions about the true cause of schistosomiasis were also present amongst interviewed schoolchildren who believed witchcraft and mosquitoes were causes of intestinal schistosomiasis. Such misconceptions may be a hindrance to implementation of a successful control program; therefore they need to be clarified before launching an integrated control program in the area. Similar to previous studies [11, 27], misconceptions about the true mode of transmission were held by many children in these communities, with beliefs that schistosomiasis could be transmitted by drinking unboiled water; walking barefoot, and shaking hands. As many inhabitants of these areas use lake water for domestic purpose including drinking and they do suffer recurrent acute water-borne infections, which may have prompted them to believe that intestinal schistosomiasis could be transmitted by drinking unboiled lake water [27].

Despite high rates of having heard about schistosomiasis, only 39.9% of the respondents reported knowing the symptoms of schistosomiasis. Low level of awareness on the signs and symptoms for intestinal schistosomiasis were reported in Siphofaneni area in the Lowvelds of Swaziland [24]. In this current study, the majority mentioned stomach ache to be a symptom for intestinal schistosomiasis contrary to blood in stool which was the most commonly reported symptom associated with intestinal schistosomiasis in western Côte d’Ivoire [26].

Despite the majority of the respondents knowing that avoiding swimming in lake water may be preventive for schistosomiasis, visiting the lake was a common practice amongst study participants due to dependency on the water for domestic and economic use including fishing, swimming, washing utensils, drinking, cooking, and watering animals. Similar results were also reported in western Kenya [11]. Misconceptions on proper preventive practices against intestinal schistosomiasis were common among study participants, including avoiding drinking unboiled lake water and washing fruits before eating. This was a misconception likely rooted in such preventive measures applying to other water-borne infections which are also endemic in the area. The observed knowledge gap on the signs, symptoms, and preventive measures against intestinal schistosomiasis among study respondents indicates lack of appropriate health education targeting this at-risk group which should be provided in combination with mass treatment campaigns. Such campaigns should enhance children’s knowledge and therefore influence positive practices which will lower re-infection rates following mass drug administration campaigns.

Respondents in this study consider fishermen and schoolchildren to be the most at risk groups for schistosomiasis. These two groups were also perceived to be the most at risk groups in a different study [11]. Despite high knowledge on the mode of transmission of intestinal schistosomiasis and the reported high rate of toilet ownership, indiscriminate defecation practices were common among study participants. This practice implies that the knowledge on the mode of transmission for intestinal schistosomiasis has not influenced children’s sanitation (toileting) practices. This finding may signify that behavioural changes, which are often more difficult to achieve, are not guaranteed by awareness alone, and that long intervals are required to ensure uptake and compliance of healthier practices [13, 28]. Similar findings have been reported elsewhere, children felt comfortable defecating close to lake water when at the lake, as it appeared inconvenient to go back home just to answer a call of nature while there was water around to clean themselves thereafter [11, 13, 23]. In another study participants reported that, in some cases where the toilets were present, people still preferred defecating in the bush where they found to be more comfortable as compared to pit latrines that were feared to house snakes and were often full [11]. These findings suggests that provision of toilets alone is not enough to eliminate the indiscriminate defecation practices, providing public education on the importance of properly using toilets in the control of schistosomiasis and other parasitic infections needs to be emphasized in a manner which reaches the targeted populations [13].

The study further revealed that toilet ownership was lower in Busanga than Kibuyi village and more respondents reported to have indiscriminate defecation practice in Busanga than was reported in Kibuyi village. This observation is likely related to the proximity of Kibuyi village to Musoma municipality which is the headquarters for Mara region potentially leading to people having better access to health information than people at Busanga village which is more distal from the larger centre. Location of the household has been found to be a significant factor in the access and utilization of toilets [29].

## Conclusion and recommendation

This study found that most schoolchildren in two villages of Rorya districts were familiar to schistosomiasis with majority mentioning schools to be the source of schistosomiasis knowledge. Despite this high rate of awareness about schistosomiasis many children had misconceptions about the true cause, mode of transmission, symptoms, and preventive measures for intestinal schistosomiasis. Thus, an appropriate health education intervention and community mobilisation are recommended in order to enhance schistosomiasis prevention and inculcate a better knowledge onto schoolchildren regarding its transmission and prevention. For an effective and successful control program against schistosomiasis, there is a need for provision of proper health education to the most at-risk groups who serve both as the main source of infection and victims for the high disease burden.

## Abbreviations

NIMR: National Institute for Medical Research
NTD: Neglected Tropical Diseases
